# NK Cells Modulate Dendritic Cell (DC) Signaling Pathways and DC Recruitment in Chlamydial Infection

**DOI:** 10.3390/ijms26083769

**Published:** 2025-04-16

**Authors:** Xinting Wang, Chunyan Zhang, Yongci Zhang, Shuhe Wang, Rony Thomas, Xi Yang

**Affiliations:** 1 Department of Immunology, University of Manitoba, Winnipeg, MB R3E 0T5, Canada; xinting.wang1@umanitoba.ca (X.W.);; 2 Department of Biochemistry and Molecular Biology, Department of Immunology, School of Basic Medical Science, Tianjin Medical University, Tianjin 300070, China; 3 State Key Laboratory of Experimental Hematology, Key Laboratory of Cellular and Molecular Immunology, Key Laboratory of Immune Microenvironment and Disease (Ministry of Education), The Province and Ministry Co-Sponsored Collaborative Innovation Center for Medical Epigenetics, Tianjin Medical University, Tianjin 300070, China

**Keywords:** *Chlamydia*, mice, natural killer cell, dendritic cell, signaling, migration, CCR5

## Abstract

Previous studies have demonstrated the significant impact of NK cells on adaptive immune responses against chlamydial infections through modulating DCs, yet the molecular mechanisms remain incompletely understood. This study investigates the role of NK cells in modulating DC signaling pathways and the recruitment of DCs during *Chlamydia muridarum* infection. Transcriptomic analyses revealed significant downregulation of key genes in DCs from NK-depleted mice involved in type I immunity, including *IL12rb2, IL-18rap*, and chemokine signaling components such as *Ccl3*, *Ccl5*, and *Ccr5.* Gene ontology (GO) analyses confirmed impaired chemokine–chemokine receptor interactions in DCs from NK-depleted mice. Moreover, flow cytometry analysis showed that NK-cell depletion reduced CCR5 expression on splenic and pulmonary DCs, impairing their migration toward CCL3 and CCL5. Furthermore, IFN-γ enhanced CCR5 expression on the surface of DCs, consequently promoting their migration, which was blocked by anti-IFN-γ antibodies. In vitro migration assays showed that treatment of DCs with IFN-γ increased their responsiveness to CCL3 and CCL5, the ligands of CCR5. Collectively, this study provides new insights into the indispensable role of NK cells in orchestrating DC signaling and the recruitment of DCs during chlamydial infection.

## 1. Introduction

*Chlamydia* is a notable intracellular bacterial pathogen that primarily infects mucosal epithelial surfaces, causing a range of diseases in humans and animals [1,2,3,4]. Protective immunity against intracellular bacterial infections is heavily reliant on a robust Th1 immune response, especially the production of IFN-γ [5,6]. NK cells, as early responders to intracellular bacterial infections, exert cytotoxic effects on infected cells and secrete cytokines, which can inhibit bacterial growth [7,8,9]. The cytokines produced by NK cells not only contribute to direct pathogen control but also influence the functionality of DCs to direct T cell responses, thus bridging innate and adaptive immunity [10,11]. The influence of NK cells on DCs may be mediated through direct cell–cell interactions and soluble factors, enabling NK cells to promote DC maturation and cytokine production [12,13,14]. NK cell activation involves signaling through receptors such as NKp46, NKG2D, and CX3CR1, which recognize ligands on DCs and infected cells [9,15].

The modulating effect of NK cells on DC functions has been demonstrated in various intracellular infections [16,17,18,19]. NK cells can enhance CD8 T cell responses in *Cytomegalovirus* infection [20] and type 1 immunity in *Plasmodium chabaudi* AS infection [20] through its modulating effect on DCs. Activated NK cells release profound amounts of IFN-γ and TNF-α, which promote DC maturation and the secretion of cytokines such as IL-12, IL-6, IL-27, IL-21, IL-23, and TGF-β by DCs. These cytokine signals provided by DCs in turn promote the adaptive immune responses against infections, particularly Th1/Th17 immunity [15,20,21,22]. Using a model of *Chlamydia muridarum* (Cm) infection, we found that NK cell depletion results in more severe disease in mice and failure to clear the pathogen [20]. The NK-depleted mice showed less mature DCs and lower levels of Th1(IFN-γ) and Th17(IL-17), but higher levels of Th2(IL-4) cytokines [20]. The other studies also reported that NK cells can promote protective type I immune responses to *Chlamydia pneumoniae* by inducing enhanced IFN-γ-producing Th1 cells and IL-17-producing Th17 cells, which were correlated with enhanced IFN-γ, IL-12, IL-17, and IL-22 production, together with T-bet and RORgt expression [23,24,25,26].

Although the effect of NK cells on the phenotype and cytokine production of DCs has been well documented in various infections, the changes in DCs regarding their signaling pathways related to function are not fully understood. In addition, although our previous studies indicate that NK cells protect mice from Cm infection through interactions between DCs and T cells [20], the molecular mechanisms underlying NK cell-mediated regulation of DC recruitment in the context of Cm infection remain unclear. In this study, we address these knowledge gaps by focusing on two primary objectives. First, we aim to map how NK cells modulate DC signaling pathways during Cm infection, by comprehensive profiling of alterations in DC gene expression related to its function. In particular, we apply GO enrichment analysis to investigate the functional implications of genes differentially expressed in DCs from NK-depleted mice. By identifying GO terms within a set of differentially expressed genes, we are able to show important functional pathways related to type 1 immunity and chemotactic changes in DCs from NK-depleted mice during chlamydial infection. Second, we investigate the contribution of NK cells to DC recruitment, particularly the involvement of CCR5 expression on DCs and its ligands. The data offer new insights into NK/DC interactions and their critical role in immune defense against chlamydial infection.

## 2. Results

### 2.1. NK Cell Depletion Exacerbates Chlamydial Infection in the Lung

**Figure 1:**
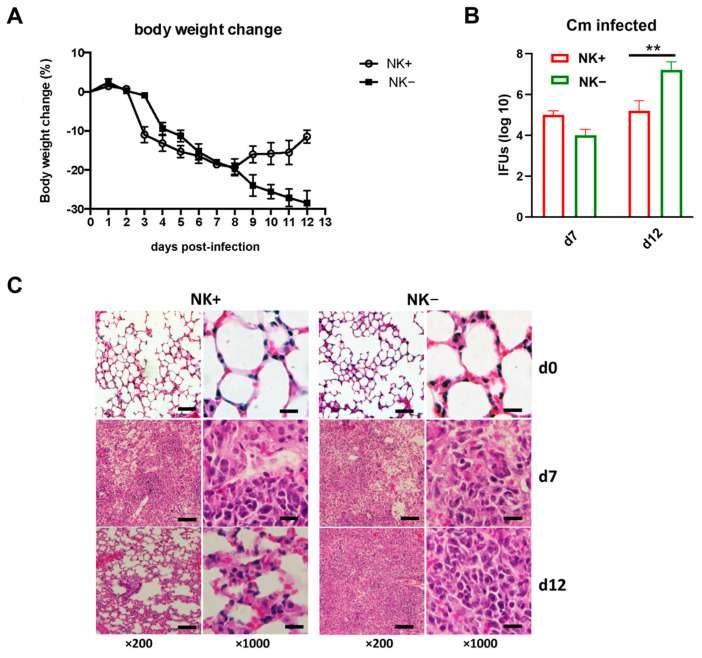
NK cell depletion leads to reduced ability to clear *Chlamydia muridarum* lung infection. C57BL/6 mice (four mice per group) were treated with control IgG (NK+) or anti–asialo-GM1 (NK-) Ab before *Chlamydia muridarum* (Cm) lung infection, as described in Materials and Methods. (**A**) More body weight loss after chlamydial infection in NK cell-depleted mice (NK-). Mice were monitored daily for body weight changes. Each point represents the mean ± SD of four mice. (**B**) Mice were sacrificed on day-7 or day-12 p.i., and the lungs were collected and analyzed for in vivo chlamydial growth, as described in Materials and Methods. (**C**) Lung sections were stained by H&E for histological analysis under light microscopy at day-7 (d7) and day-12 (d12) p.i. One representative experiment of three independent experiments with similar trends is shown. Magnification level is shown at ×200 (scale bar, 200 μm) or ×1000 (scale bar, 40 μm). ** *p* < 0.01.

To confirm the functional role of NK cells in host defense against Cm infection, which was reported in previous reports [9,20], NK cell-depleted mice (NK-) and sham IgG antibody-treated NK-intact C57BL/6 mice (NK+) were intranasally infected with Cm, and the infection outcomes were assessed. Results revealed that NK cell depletion significantly worsened the infection progression. NK-depleted mice experienced more severe and persistent body weight loss compared to NK-intact controls (Figure 1A). This more severe body weight loss was accompanied by a notably higher bacterial load in the lungs of NK-depleted mice at the later stages of infection (day 12) relative to sham-treated mice (Figure 1B). Lung histological analysis further demonstrated substantially greater tissue damage and pathological changes in the NK-depleted mice (Figure 1C). The data confirm the important role of NK cells involving host defense against Cm lung infections.

### 2.2. Transcriptional Alterations in DCs of NK-Depleted Mice During Chlamydial Infection

**Figure 2:**
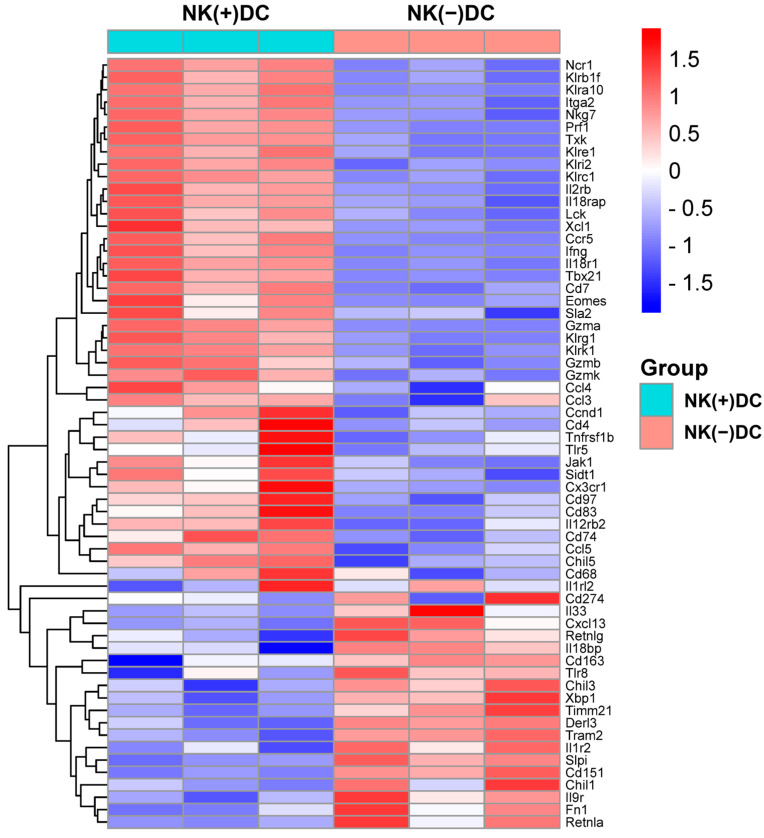
Heat map of differentially expressed mRNAs in microarray analyses of NK(+)DC and NK(−) DC isolated from Cm-infected mice. Splenic DCs were isolated from mice NK-intact (NK(+)DC) and NK-depleted (NK(−)DC) mice at three days post-infection (*n* = 3 mice/group). The total RNA of the DCs was extracted, and GeneChips were scanned by Affymetrix for microarray analysis. Differentially expressed genes were identified by ANOVA (fold-change ≥1.5; *p* < 0.05). The heat map was generated by SRplot (https://www.bioinformatics.com.cn, accessed on 4 October 2022).

Since previous reports have identified that the protective role of NK cells in chlamydial infection largely relies on its role in modulating DCs [12,13], we here focused on the impact of NK cells on DC signaling pathways. We first conducted a microarray analysis to examine the gene expression profiles in DCs isolated from NK-intact [NK(+)DC] and NK-depleted mice [NK(-)DC] following Cm infection. Splenic DCs were isolated from mice three days post-infection. Total RNA was extracted from both NK(+)DC and NK(-)DC populations for transcriptomic analysis. Using a stringent differential expression threshold (≥1.5 fold-change, *p* < 0.05), multiple differentially expressed genes (DEGs) were identified. Notably, the majority of the tested genes were found to be downregulated in the NK(-)DC group relative to the NK(+)DC counterpart, as visualized in the heat map (Figure 2).

In particular, NK(-)DC showed significantly downregulated *Ifng*, *Ccl3*, *Ccl4*, *Ccl5*, *Xcl1,* and *Ccr5*, indicating impaired type I immune responses and reduced chemotactic signals necessary for DC migration and recruitment to inflamed tissues. Additionally, genes encoding granzymes and perforin (e.g., *Gzma, Gzmb, Gzmk, Prf1*), essential for NK-mediated cytotoxicity and DC activation, were significantly reduced. Consistently, transcriptional regulators T-bet (*Tbx21*) and Eomes, which are pivotal for Th1 differentiation and IFN-γ production, were also suppressed. The reduced expression of signaling molecules for cytokine receptors like *Il12rb2* and *Il18r1*, which are critical for DC responsiveness to IL-12 and IL-18, indicates an additional layer of functional impairment in the development of type I immune responses. DCs maturation markers such as *Cd83*, *Cd74*, and migration regulators (*Cd97, Sla2*) were also downregulated. Together, the microarray analysis reveals significant defects of NK(-)DCs in maturation and migration to infection sites (Figure 2).

**Figure 3:**
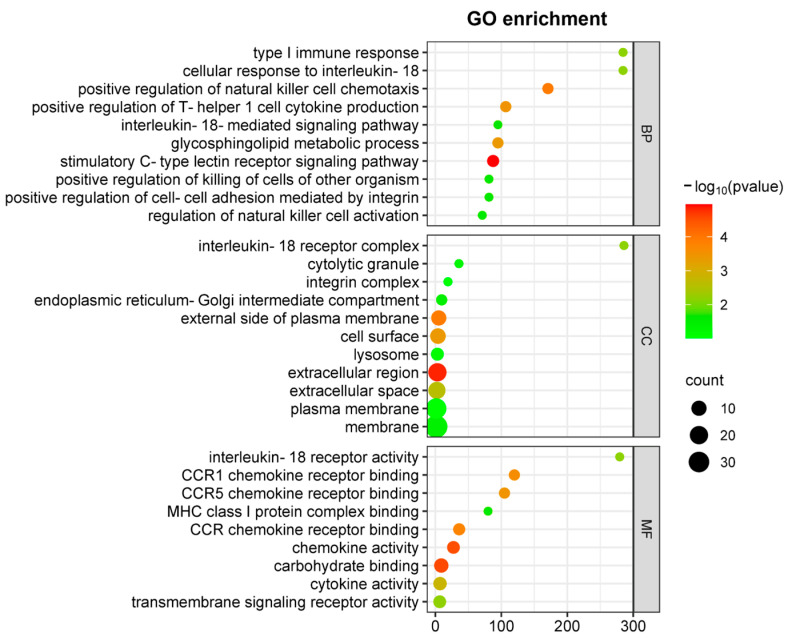
GO enrichment analysis of DEGs in comparison between NK(+)DC and NK(-)DC post-infections. Gene ontology (GO) enrichment analysis of differentially expressed genes (DEGs) are mapped by microarray in NK(+)DC and NK(-)DC post-infection samples. Dot plots show enriched GO terms in the categories of biological process (BP), cellular components (CC), and molecular function (MF). The analysis was performed using SRplot (https://www.bioinformatics.com.cn, accessed on 15 October 2022) with a significance threshold of adjusted *p*-value < 0.05. Dot size represents the number of genes associated with each term, and the color intensity indicates the significance of enrichment.

Moreover, functional categorization of DEGs through GO analysis (Figure 3) revealed significant enrichment in NK(+)DC biological processes associated with type I immune responses following chlamydial infection. These processes include Th1-type cytokine production and IL-18-mediated signaling pathways in cellular components involved in the IL-18 receptor complex, and in molecular functions involved in chemokine receptor activity. Within the top enriched GO terms, downregulation of key cytokines/chemokines, including *Ccl3, Ccl4, Ccl5*, and signaling molecules such as *Il12rb2* and *Il18r1*, points to a defect of NK(-)DC in the recruitment and the activation of Th1 immune responses.

**Figure 4:**
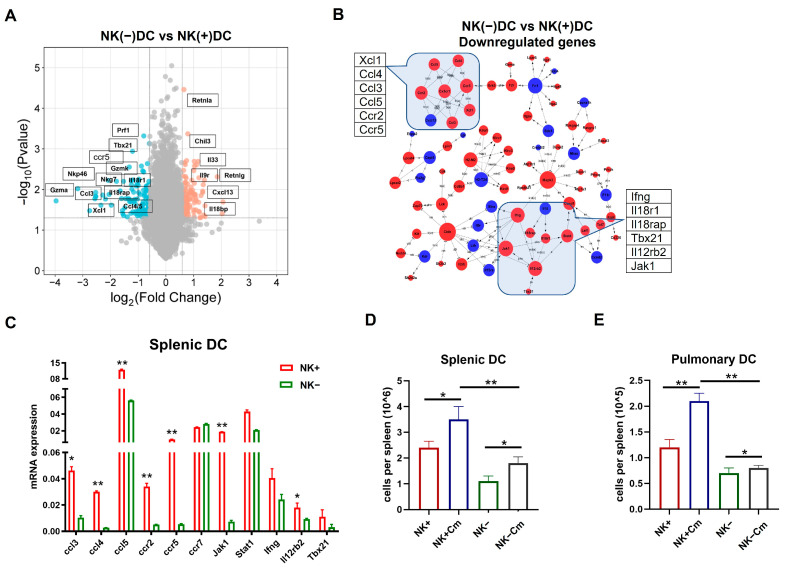
NK cell-depletion reduces DC recruitment and modulates key immune pathways on DCs during chlamydial infection. (**A**) Volcano plot showing the differentially expressed genes mapped by microarray in NK(+)DC and NK(-)DCs post-infection samples. The *x*-axis represents the log2 fold-change (log2FC), and the *y*-axis represents the −log10 adjusted *p*-value. Red and blue dots indicate significantly upregulated and downregulated genes, respectively. Non-significant genes are shown in gray. (**B**) BIOCARTA pathway analysis of key differentially expressed genes generated using the DAVID database (www.biocarta.com, accessed on 5 November 2022). (**C**) qRT-PCR analysis of mRNAs in NK(+) and NK(-) DCs from mice three days post-infection. (**D**,**E**) Absolute cell counts of total splenic and pulmonary DCs across four experimental groups (NK-intact and NK-depleted mice with or without Cm infection). Cm indicates chlamydial infection at three days post-infection. Significant differences between the treated and control groups are denoted by * *p* < 0.05 and ** *p* < 0.01.

Volcano plot analysis (Figure 4A) revealed a substantial downregulation of genes in NK(-)DC compared to NK(+)DC, including key regulators of type I immune responses such as *Prf1*, *Gzmk*, *Gzma*, *Tbx21*, *Nkp46*, and *Nkg7*. Genes associated with the IL-18 receptor complex (*Il18r1*, *Il18rap*) and chemokine receptor binding (*Ccl3*, *Ccl4*, *Ccl5*, *Ccr5*, *Xcl1*) were also significantly downregulated. These results suggest a critical impairment in NK(-)DC functionality, particularly in the signaling pathways governing cytotoxicity, chemotaxis, and inflammation. DAVID database analysis (Figure 4B) supported these findings, with genes clustering into pathways such as chemokine signaling (*Ccl3*, *Ccl5*, *Ccr5*) and type I immune responses. The clustering pattern further highlights the reliance of DCs on NK-derived signals for activation and migration during Cm infection. Together, the novel gene profiling and signaling pathway data at the molecular level confirms the significant modulating effect of NK cells on the cellular function of DCs in promoting type I immunity and the potential role for DC recruitment.

### 2.3. NK-Depletion Reduces DC Recruitment and Alters Key Immune Pathways on DCs in Chlamydial Infection

On the basis of a comprehensive analysis of signaling pathways by microarray that showed significant changes in the genes related to the development of type 1 immunity and the migration of cells in NK(-)DCs, we performed quantitative reverse transcription PCR (qPCR) on the some of the identified differentially expressed genes to validate the microarray results. The qPCR data demonstrated a strong correlation with the microarray findings. Specifically, genes associated with the CCR5-Ccl3/4/5 chemokine signaling exhibited significant downregulation in NK(-)DC compared to NK(+)DC. Similarly, the critical regulator of type I immune response was significantly downregulated in the qPCR analysis (Figure 4C).

We subsequently focused on the functional impact of the alteration of genes related to DC migrations and recruitments. The rationale for strategically targeting migration was that our previous work had already found a significant functional defect of NK(-)DC in inducing protective type 1 immunity, while the functional effect of NK on DC migration has not been addressed in chlamydial infection. To verify the functional relevance of the observed changes in chemokine signaling pathways, we assessed DC recruitment in NK-intact and NK cell-depleted mice after Cm infection. We found that, three days post-infection, DC recruitment to the lungs and spleen was significantly reduced in the NK-depleted mice than the NK-intact controls (Figure 4D,E). These results confirm the impact of NK cells on efficient DC migration and recruitment to infection sites.

### 2.4. NK Cells Enhance CCR5 Expression on the Surface of DCs and the DC Migration Depends on CCL3/5-CCR5 Interaction During Chlamydial Infection

**Figure 5:**
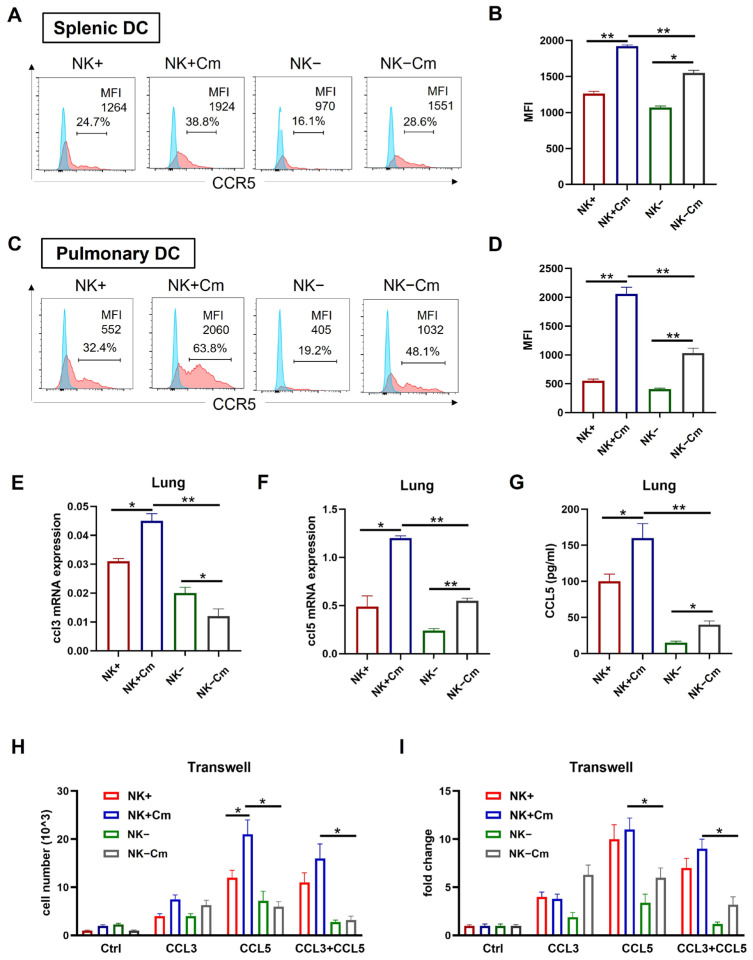
NK cells promote CCR5 expression on DCs and the DC migration depends on CCL3/5-CCR5 interaction during chlamydial infection. (**A**–**D**) To delete NK cells, mice were intraperitoneally injected with 30 μL anti-asialo-GM1 every two days, starting three days prior to intranasal infection. The control group was treated with normal rabbit IgG following the same protocol. On day-3 post-infection, spleen and lung single-cell suspensions were prepared and DCs were gated as CD11c+ I-A/E+ and F4/80- cells. CCR5 expression on these cells was analyzed via flow cytometry. The mean fluorescence intensity (MFI) for each group is shown. (**A**) Flow cytometry of splenic DC; (**B**) summary data for splenic DC; (**C**) flow cytometry of pulmonary DC; (**D**) summary data for pulmonary DC. One representative of three independent experiments with four mice in each group is shown. (**E**,**F**) mRNA levels of CCL3 and CCL5 in the lung tissues across four experimental groups (NK-intact and NK-depleted mice with or without Cm infection). Statistical significance between treated and control groups is represented as * *p* < 0.05 and ** *p* < 0.01. (**G**) CCL5 production in the lung tissues across the four experimental groups, measured by ELISA; * *p* < 0.05, ** *p* < 0.01. (**H**,**I**) Freshly isolated splenic DCs across the abovementioned four experimental groups were tested for migration toward CCL3 and CCL5 (100ng/mL) using a transwell assay. After 4 h incubation, cells in the lower chamber were collected, and counted under a microscope. Both absolute values and fold changes are presented. Fold change in the Ctrl group is normalized to 1 as the baseline (**I**). Data are presented as mean ± SD, representing three independent experiments with at least three mice in each group in an independent experiment. * *p* < 0.05; ** *p* < 0.01. Cm indicates as chlamydial infection at three days post-infection.

CCR5 is a receptor closely associated with DC recruitment and migration to lymph nodes and infection sites [27,28,29,30,31]. On the basis of gene profiling showing the reduction in the CCR5 signal pathway in NK(-)DC, we further analyzed CCR5 expression on splenic and pulmonary DCs. Flow cytometry analysis showed a significant increase in CCR5 on the surface of splenic (Figure 5A,B) and pulmonary (Figure 5C,D) DCs following chlamydial infection. However, the increase in CCR5 in the DCs from NK-depleted mice was much less than that in the NK-intact mice following the infection, especially in pulmonary DCs (Figure 5A–D).

We further tested the levels of CCL3 and CCL5, the natural ligands for CCR5 in the tissues of Cm infected mice. We examined their expression in spleen and lung tissues from infected NK-depleted (NK-) and NK-intact (NK+) mice, as well as from sham-treated controls, using qPCR and ELISA, respectively. The results showed elevated *Ccl3* and *Ccl5* mRNA levels in the lungs of infected NK-intact mice relative to uninfected controls. However, NK cell depletion significantly reduced *Ccl3* and *Ccl5* expression in the lungs of infected mice compared to NK-intact controls (Figure 5E,F). Furthermore, ELISA results showed significantly lower CCL5 production in the lung tissues of NK-depleted mice than in NK-intact mice (Figure 5G). These findings suggest that NK cell depletion impairs CCL3 and CCL5 production in the tissues of infection.

To directly assess the effect of NK cells on DC migration toward CCL3 and CCL5, we conducted a transwell migration assay using DCs isolated from Cm-infected NK-intact and NK-depleted mice. The assay demonstrated higher migration of DCs in NK-intact (NK+) mice showing significantly greater migratory responses to CCL3 and CCL5 than DCs from NK-depleted mice (Figure 5H). Collectively, these results indicate that NK cell depletion reduces CCR5, CCL3, and CCL5 levels, consequently impairing DC recruitment and migration during Cm infection.

### 2.5. NK Cell-Produced IFN-γ Promotes CCL3/5-CCR5-Dependent DC Recruitment During Chlamydia Lung Infection

**Figure 6:**
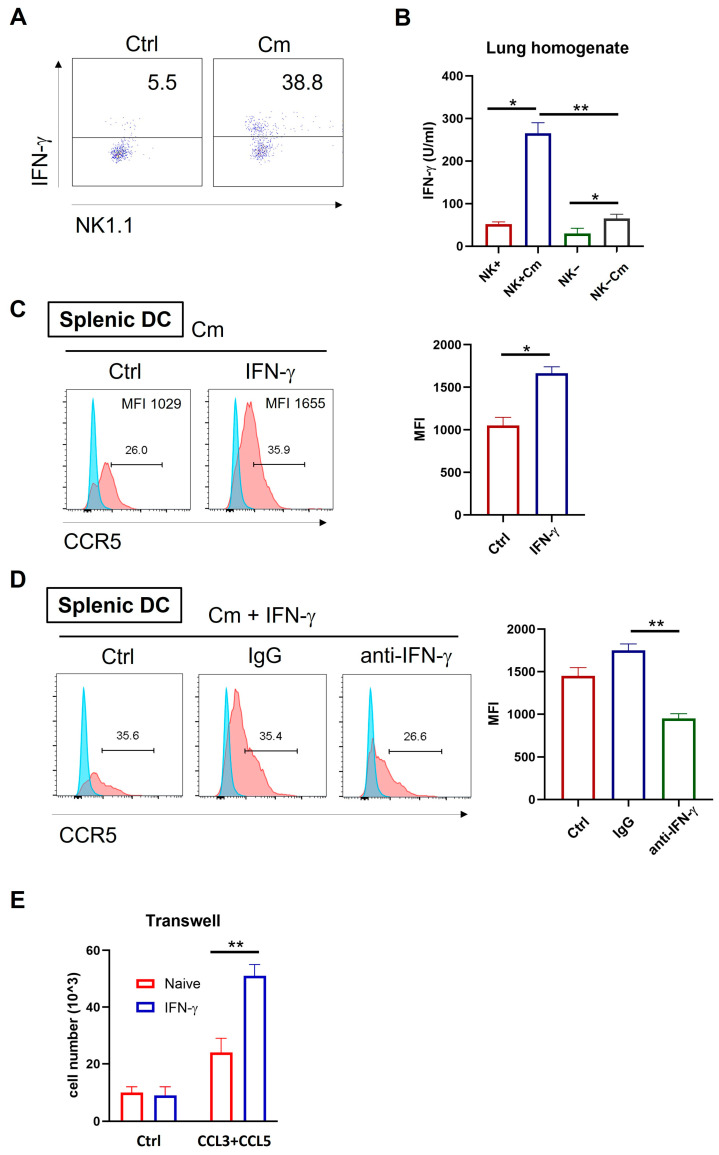
NK cell-produced IFN-γ enhances CCL3/5-CCR5-dependent DC recruitment during chlamydial infection. (**A**) Wild-type C57BL/6 mice were infected intranasally with Cm and the lung tissues were harvested at three days post-infection. Single-cell suspensions were prepared and the NK cells were isolated using anti-NK magnetic beads. Intracellular IFN-γ expression on gated NK cells (CD3-NK1.1+) was assessed by flow cytometry following in vitro activation with a PMA cocktail for 4 h. (**B**) IFN-γ production in lung tissues across four experimental groups (NK-intact and NK-depleted mice with or without Cm infection). The lung tissues were homogenized and centrifuged and the cytokine levels measured by ELISA. (**C**) Splenic DCs from naïve C57BL/6 mice were cultured in vitro with 10 ng/mL IFN-γ or culture medium alone, along with Cm infection. After 24 h, CCR5 expression was assessed by flow cytometry. The summary data for fluorescence intensity (MFI) are shown on the right side. (**D**) Splenic DCs from naïve C57BL/6 mice were cultured in vitro with 10 ng/mL IFN-γ, along with Cm infection, in the presence of 50 μg/mL anti-IFN-γ antibody (anti-IFN-γ) or isotype-control antibody (IgG) or no antibody being added (Ctrl). After 24 h, CCR5 expression was assessed by flow cytometry. The mean fluorescence intensity (MFI) in each condition is summarized on the right side. (**E**) Migration of splenic DCs toward CCL3/5 was assessed in response to IFN-γ stimulation using a transwell assay. Briefly, splenic DCs from naïve C57BL/6 mice were cultured in vitro with CCL3 plus CCL5 (100 ng/mL) in the presence or absence of 10 ng/mL IFN-γ. After 4 h incubation, cells in the lower chamber were collected and counted under a microscope. Data are presented and shown as mean ± SD for each group, with at least three mice per group and one representative from three independent experiments. * *p* < 0.05; ** *p* < 0.01.

Previous studies have linked IFN-γ-producing NK cells with protective immune responses during chlamydial infection, mainly through enhancement of Th1 immunity [7,20]. To explore if the NK-mediated enhancement in DC migration is also related to IFN-γ, we examined IFN-γ production by NK cells and the effect of IFN-γ on CCR5 expression and cell migration during chlamydial lung infection. Intracellular cytokine analysis of freshly isolated pulmonary cells showed significantly higher IFN-γ levels in pulmonary NK cells (CD3-NK1.1+) from infected mice compared to naïve controls (Figure 6A). In addition, the lung homogenates from infected or control mice were also analyzed by ELISA. The result revealed that NK cell depletion significantly reduced IFN-γ production in the local tissues compared to NK-intact mice (Figure 6B).

Next, we investigated the effect of IFN-γ on CCR5 expression in Cm-infected DCs. Splenic DCs from naïve WT mice were cultured in vitro with IFN-γ or culture medium only, along with Cm infection. After 24 h, CCR5 expression on the DCs was assessed by flow cytometry. CCR5 levels were significantly increased due to the presence of IFN-γ in the culture in both percentage of positive cells and fluorescent density (Figure 6C). Remarkably, this enhancement effect is virtually blocked by anti-IFN-γ antibodies (Figure 6D).

To functionally determine if IFN-γ affects DC migration via CCR5 and CCL3/CCL5 axis, we isolated splenic DCs from naïve mice and cultured them with the chemokines CCL3 and CCL5 in the presence or absence of IFN-γ stimulation. Migration assays showed that IFN-γ-stimulated DCs had significantly increased migratory capacity toward CCL3 and CCL5 compared to controls (Figure 6E). Together, these findings suggest that NK cells can modulate DC migration through enhanced CCR5 expression via IFN-γ production.

## 3. Discussion

This study addressed the modulating effect of NK cells on DCs during chlamydial infection, with a specific focus on NK cell-mediated regulation of DC signaling and recruitment. Our results underscore the pivotal role of NK cells in shaping DC-mediated immunity through cytokine signaling and chemokine receptor modulation. Using NK cell-depleted mice, we observed significant alterations in DC gene expression profile related to its functional capacity, providing molecular evidence that NK cells modulate DC’s ability to initiate and sustain type I immune responses. Moreover, we found that the CCR5/CCL3 and CCL5 chemokine axis plays an important role in promoting DC migration/recruitment in response to NK-derived signals. The results of gene signaling align with previous studies showing the importance of NK cells in orchestrating protective immune response to chlamydial pathogens by promoting DC function [32] and extend our understanding on the role of NK cells to modulate DC recruitment. The findings fill a critical knowledge gap regarding the comprehensive molecular profile of DCs that are influenced by NK cells during chlamydial infection.

An intriguing aspect of our study is the role of NK cells in shaping DC functionality through various signaling pathways. DC activation is driven by various cytokines critical for protective immunity, including IL-12, IL-18, and IFN-γ, which promote Th1 responses, and IL-23 and IL-17, which drive Th17 responses [33]. IL-12, secreted by activated DCs, is a master regulator of NK cell activation and T cell differentiation [34]. It amplifies IFN-γ production by NK cells, creating a feedback loop essential for Th1 immunity [5,33]. IL-18 complements this loop by enhancing the cytotoxicity of NK cells and augmenting IFN-γ secretion. This coordinated response ensures efficient DC maturation, antigen presentation, and T cell activation. Consistently, our transcriptomic analysis shows significant downregulation of genes involved in the IL-12 and IL-18 signaling pathways in DCs from NK cell-depleted mice with Cm infection. These include key regulators such as *Il12rb2*, which encodes the IL-12 receptor β2 subunit, and *Il18rap*, which encodes the IL-18 receptor accessory protein. These results suggest a diminished capacity of DCs to respond to these cytokines in the absence of NK cell-derived signals. The impaired expression of these signaling pathways likely disrupts the positive feedback loop between IL-12, IL-18, and IFN-γ, resulting in reduced DC activation and subsequent T cell priming. The findings demonstrate the integral role of NK cells in the initiation and maintenance of DC-driven Th1 immunity during chlamydial infection. In contrast, type 2 immune responses have been reported to be associated with impaired pathogen clearance and pathology in the lung [35,36]. In line with the previous findings, our gene profiling on DCs upon NK depletion under Cm infection also indicates the upregulated genes related to type II cytokines and signaling such as *Il33*, *Il9b*, and *Il18bp*.

Our findings provide strong evidence that NK cells orchestrate DC function by influencing DC migration and recruitment. The data revealed that NK cell-mediated modulation of CCR5 expression on DCs, along with the production of chemokines CCL3 and CCL5 in the lungs, contributed to DCs recruitment to infection sites. More interestingly, we demonstrate that IFN-γ is critical for upregulating CCR5 expression on DCs, facilitating their migration to the sites of infection. In vivo, the IFN-γ is more likely produced by NK cells because they are the major IFN-γ producers in the early stage of infection, although it could be produced by other cells. The data showing that the IFN-γ production can influence the expression of CCR5 on the DCs (Figure 6C,D) and consequently promote DC migration extend our understanding of the role of NK cells in chlamydial infection and the effect on DCs. Our microarray analysis corroborates this observation by demonstrating the significant downregulation of genes associated with chemokine receptor binding and type I immune responses in NK-depleted DCs. The ability of NK cells to produce IFN-γ was shown to be critical for promoting DC maturation and antigen presentation, supporting robust Th1 polarization [37,38]. This is consistent with earlier studies that identified IFN-γ as a key regulator of DC-mediated immunity, particularly through its enhancement of IL-12 and IL-18 secretion by DCs. The molecular pathways identified here, including the role of NK cells in regulating chemokines such as CCL3, CCL4, and CCL5 together with their receptor CCR5 expression by DCs may be relevant to other infectious and inflammatory conditions. For instance, NK/DC crosstalk has been implicated in viral and parasitic infections [39,40,41], where similar mechanisms of cytokine signaling and chemokine receptor regulation could be involved in shaping immune responses. Exploring these parallels could provide valuable insights into the common principles governing NK/DC interactions across different disease contexts. Moreover, although our analysis here mainly focused on the role of IFN-γ, the other factors involved with NK cells or other cell types may also contribute to the observed effect. Furthermore, the current study focuses on the analysis of splenic DCs. In the future, it would be important to study pulmonary DCs, which are in the local area of infection. Furthermore, the potential for differential expression of CCR5 on different DC subsets should also be tested, and the contribution of CCR5 could be confirmed by other approaches, such as inhibiting its expression using inhibitors. It should also be mentioned that the present study focused on the role of NK cells in modulating DC function, thus consequently promoting T cell immunity, rather than the role of NK cells per se in the control of chlamydial infection, which is more likely to happen during the very early stage—hours or days following the infection.

In conclusion, our study demonstrates a profound modulating effect of NK cells on DC signaling related to the development of protective immunity and DC recruitment. The findings underscore the importance of understanding the intricate interactions between innate immune cells, which can establish the way toward bolstering immune responses to chlamydial and potentially other intracellular bacterial pathogens.

## 4. Materials and Methods

### 4.1. Animals

C57BL/6 mice used in this study were bred at the University of Manitoba’s breeding facility. All experiments utilized female mice between 6 and 7 weeks of age. The research was conducted in accordance with the Canadian Council on Animal Care guidelines, and all procedures were approved under protocol number 15-008 by the Protocol Management and Review Committee at the University of Manitoba Bannatyne Campus.

### 4.2. Chlamydia Infection Mice Model

The culture and propagation of Cm followed previously established protocols [9]. In brief, Cm was maintained in HeLa 229 cells using Eagle’s minimum essential medium (MEM) supplemented with 10% fetal bovine serum (FBS) and 2 mM L-glutamine over a 48 h period. Chlamydia elementary bodies were purified, quantified, and stored at −80 °C in a sucrose–phosphate–glutamic acid (SPG) buffer until use. For infection, mice were lightly sedated with isoflurane and intranasally inoculated with 1 × 10^3^ inclusion-forming units (IFUs) of Cm in 40 μL of SPG buffer. Control mice received the same volume of SPG buffer without Cm.

### 4.3. NK Depletion Mice Model

To deplete NK cells in vivo, mice were injected intraperitoneally with polyclonal anti-asialo-GM1 (Wako, Japan). Treatment consisted of 30 μL anti-asialo-GM1 or an equivalent volume of normal rabbit IgG (control) diluted in 30 μL phosphate-buffered saline (PBS). Injections were administered every 48 h, beginning three days prior to Cm infection. Flow cytometry analysis confirmed depletion efficiency exceeding 95%.

### 4.4. Isolation of Pulmonary Cells

Mice were sacrificed aseptically at designated time points after intranasal Cm infection. Lungs were excised, finely minced, and enzymatically digested at 37 °C for 1 h in a solution containing 2 mg/mL collagenase XI and 100 μg/mL DNase I (both sourced from Sigma-Aldrich, St. Louis, MO, USA). The resulting suspension was passed through 70 μm strainers to remove tissue debris, followed by red blood cell lysis using ACK buffer (Thermo Fisher Scientific, Waltham, MA, USA). Cells were washed twice in MACS buffer before being resuspended in culture medium for flow cytometry or further assays. For in vitro stimulation, cells were cultured at a density of 7.5 × 10⁶ cells/mL with or without UV-inactivated Cm (10⁵ IFU/mL) in RPMI 1640 medium supplemented with 10% heat-inactivated FBS, 25 μg/mL gentamicin, 2 mM L-glutamine, and 5 × 10⁻⁵ M 2-mercaptoethanol. After 72 h, culture supernatants were collected, and IFN-γ levels were quantified using an ELISA kit with BD Pharmingen (San Jose, CA, USA) antibodies for capture and detection.

### 4.5. Chemokine Measurements

Mice were sedated with isoflurane and infected intranasally with 1 × 10^3^ IFUs of Cm in 40 μL SPG buffer. At three days post-infection, lungs were harvested, homogenized in RPMI 1640 medium using a cell grinder (Vernon Hills, IL, USA), and centrifuged. The supernatant was stored at −80 °C until analysis. ELISA kits were employed to measure IFN-γ and CCL5 concentrations.

### 4.6. Chemotaxis Assay

DCs were isolated from spleens of both Cm-infected and uninfected mice. Chemotaxis assays were performed using a chamber system where the lower wells contained either CCL3 or CCL5 (100 ng/mL) diluted in chemotaxis medium (RPMI 1640 supplemented with 1% bovine serum albumin) or control medium (RPMI 1640 alone). A 50 μL suspension of DCs (1 × 10⁶ cells/mL) was introduced into the upper chamber, separated by an 8 μm pore polycarbonate filter. Following a 4 h incubation at 37 °C with 5% CO₂, filters were fixed, stained with crystal violet, and cells adhered to the underside were enumerated at 200× magnification. Additionally, cells from the lower chamber were collected, centrifuged, resuspended in RPMI 1640, and counted using a microscope (Nikon,Tokyo, Japan). Migration was quantified as cells per high-power field (HPF). Splenic DCs from naïve wild-type mice were cultured in vitro with IFN-γ (10 ng/mL, R&D) or an IFN-γ-neutralizing antibody (50 μg/mL, R&D) in the presence of Cm. After 24 h, CCR5 expression (anti-CCR5, eBioscience, San Diego, CA, USA) was evaluated via flow cytometry. Separately, naïve splenic DCs were incubated with either CCL3 or CCL5 (100 ng/mL) with or without 10 ng/mL IFN-γ.

### 4.7. Flow Cytometry

Freshly isolated single-cell suspensions were stained with fluorescent-labeled antibodies against surface markers (anti-NK1.1 or anti-CCR5, eBioscience) or isotype controls (eBioscience). Following fixation with 2% paraformaldehyde, data were collected using a BD Biosciences FACS Canto II cytometer (San Jose, CA, USA) and analyzed with FlowJo X software (v10.8.1). To assess intracellular cytokine production, cells were stimulated with PMA (50 ng/mL) and ionomycin (1 μg/mL) in the presence of brefeldin A (5 μg/mL) for 3 h, followed by intracellular IFN-γ staining (anti-IFN-γ, eBioscience, San Diego, CA, USA) and flow cytometric analysis.

### 4.8. Microarray

Mice were divided into four groups (control, NK-depleted, Chlamydia-infected, and NK-depleted + Chlamydia-infected; *n* = 3/group). Three days post-infection, spleens were collected, and DCs were purified using CD11c magnetic beads (BD Pharmingen, San Jose, CA, USA). Total RNA was extracted using TRIzol reagent (Sigma) and hybridized onto GeneChips (Affymetrix, CA, USA) for scanning. Differential gene expression was identified using ANOVA, with significance defined as a fold-change ≥1.5 and *p* < 0.05. Functional enrichment was analyzed using SRplot (https://www.bioinformatics.com.cn/, accessed on 4 October 2022) for heat maps and gene ontology, while pathway analysis was performed using DAVID (Database for Annotation, Visualization, and Integrated Discovery).

### 4.9. Quantitative RT-PCR

Total RNA from spleen DCs was extracted using TRIzol (Invitrogen, Carlsbad, CA, USA) and reverse-transcribed using the RevertAid™ First Strand cDNA Synthesis Kit (Roche, Switzerland). Real-time PCR was performed on a StepOne™ system (Applied Biosystems, Foster City, CA, USA) with SYBR Green Master Mix (Roche). Primers are listed in Table 1. PCR conditions were 95 °C for 2 min, followed by 40 cycles of 95 °C for 30 s and 60 °C for 1 min. Fold changes were calculated using the ^ΔΔ^Ct method and normalized to 18S RNA levels.

### 4.10. Statistica Analysis

Unpaired Student’s *t* test was used to assay the statistical significance in the comparison of two different groups. One-way ANOVA analysis was used for analyzing data from the experiments with multiple groups. A *p* value less than 0.05 was considered significant.

## Figures and Tables

**Table 1:** Primers sequences for qRT-PCR.

Gene	Forward	Reverse
Ccr7	TGTACGAGTCGGTGTGTCTTC	GGTAGGTATCCGTCATGGTTTG
Ccr2	ATCCACGGGCATACATACAACATC	CAAGGGCTCACCATCATGTAG
Ccr5	TTTTCAAGGGTCAGTTCCGAC	GGAAAGGACATCATGTTACCCAC
Ccl3	TTCTCTGTACCATGACACTGTGC	CGTGGAATCTTCTTCTCAGTGG
Ccl4	TTCTCGTGTTTTCCTTACACCT	CTGTCTGGCCTTTTTGTGAG
Ccl5	GCTGCTTTGCCTACCTCCTCC	TCGAGTGACAACAACAGGACTGC
Ifng	ATGAACGCTACACACTGCATC	CCATCCTTTTGCCAGTTCCTC
Stat1	GGCACTCTGGAAGTTCAGTAT	CCATCCTTGTGTTCATCACTTGC
Jak1	TGTGGAGTGGTGTTTTGCTTT	CTGCGCCCTTTACTCTCCTGTT
IL12rb2	AGAGAATGCTCATTGGCACTTC	AACTGGGAATAATGTGAACAGCC
Tbx21	AGCAAGGACGGGCAAGATGTT	GGGTGGACATATAAGCAGGGTTC
18s	AGTAACGGCGAGTCAACTGCG	CTTTGAGGTGGAAGGACAGGGAG

## Data Availability

Data is contained within the article.
